# Post-COVID changes in lung function 6 months after veno-venous extracorporeal membrane oxygenation: a prospective observational clinical trial

**DOI:** 10.3389/fmed.2023.1288679

**Published:** 2023-12-20

**Authors:** Alexandra Pálfi, Ádám L. Balogh, Gabriella Polónyi, Domonkos Schulcz, Éva Zöllei, Gábor Bari, Gergely H. Fodor, Kristóf Baráth, Attila Somfay, Ferenc Peták, Barna Babik

**Affiliations:** ^1^Department of Anesthesiology and Intensive Therapy, University of Szeged, Szeged, Hungary; ^2^Cardiac Surgery Unit, Department of Internal Medicine, University of Szeged, Szeged, Hungary; ^3^Department of Medical Physics and Informatics, University of Szeged, Szeged, Hungary; ^4^Department of Pulmonology, University of Szeged, Szeged, Hungary

**Keywords:** respiratory failure, ECMO, post-COVID-19, respiratory oscillometry, plethysmography, spirometry

## Abstract

**Background:**

Severe coronavirus disease 2019 (COVID-19) may require veno-venous extracorporeal membrane oxygenation (V-V ECMO). While V-V ECMO is offered in severe lung injury to COVID-19, long-term respiratory follow-up in these patients is missing. Therefore, we aimed at providing comprehensive data on the long-term respiratory effects of COVID-19 requiring V-V ECMO support during the acute phase of infection.

**Methods:**

In prospective observational cohort study design, patients with severe COVID-19 receiving invasive mechanical ventilation and V-V ECMO (COVID group, *n* = 9) and healthy matched controls (*n* = 9) were evaluated 6 months after hospital discharge. Respiratory system resistance at 5 and 19 Hz (R_5_, R_19_), and the area under the reactance curve (AX_5_) was evaluated using oscillometry characterizing total and central airway resistances, and tissue elasticity, respectively. R_5_ and R_19_ difference (R_5_–R_19_) reflecting small airway function was also calculated. Forced expired volume in seconds (FEV_1_), forced expiratory vital capacity (FVC), functional residual capacity (FRC), carbon monoxide diffusion capacity (DLCO) and transfer coefficient (KCO) were measured.

**Results:**

The COVID group had a higher AX_5_ and R_5_–R_19_ than the healthy matched control group. However, there was no significant difference in terms of R_5_ or R_19_. The COVID group had a lower FEV_1_ and FVC on spirometry than the healthy matched control group. Further, the COVID group had a lower FRC on plethysmography than the healthy matched control group. Meanwhile, the COVID group had a lower DLCO than healthy matched control group. Nevertheless, its KCO was within the normal range.

**Conclusion:**

Severe acute COVID-19 requiring V-V ECMO persistently impairs small airway function and reduces respiratory tissue elasticity, primarily attributed to lung restriction. These findings also suggest that even severe pulmonary pathologies of acute COVID-19 can manifest in a moderate but still persistent lung function impairment 6 months after hospital discharge.

**Trial registration:**

NCT05812196.

## Introduction

The lungs are the primary target of severe acute respiratory syndrome coronavirus 2 (SARS-CoV-2). This mechanism can partly involve the local entry of the pathogen and endothelial dysfunction caused by the large endothelial surface per unit tissue mass in the pulmonary system. Accordingly, the most evident outcomes that can determine overall patient status are commonly related to acute detrimental changes in lung function and structure. Severe gas exchange defects may require respiratory support with non-invasive or invasive mechanical ventilation. If these intensive care modalities cannot restore and maintain sufficient oxygenation, the application of veno-venous extracorporeal membrane oxygenation (V-V ECMO) can be an alternative modality for patients with severe coronavirus disease 2019 (COVID-19) (1–5).

Life-threatening adverse events commonly manifest in the acute phase of coronavirus infection (6). However, the remaining symptoms after COVID-19 recovery also present a major challenge among healthcare providers (2, 4, 5, 7–16). Similar to the acute phase, the lungs are the most persistently and extensively affected among the organs after COVID-19 infection (2, 4, 7–15). Several factors can influence the development and severity of post-COVID-19 pulmonary symptoms. These include age, pre-existing medical conditions, and the severity of infection in the acute phase (8, 17–19). The need for V-V ECMO therapy ultimately indicates the presence of extremely severe acute pulmonary dysfunction. Hence, assessing the long-term pulmonary effects of severe coronavirus infection in patients requiring V-V ECMO can identify post-COVID-19 lung functional changes.

Therefore, the current study aims to assess the long-term (6 months) pulmonary effects of severe COVID-19 requiring V-V ECMO support in the acute phase of infection.

## Materials and methods

### Participants

This prospective observational study included patients with severe acute COVID-19 who received V-V ECMO support at our tertiary center between March 2021 and May 2022 (COVID group). Assessments were performed 6 months after rehabilitation discharge. One patient in the COVID group had psoriasis, while others had no previously registered comorbidity. Patients for the control group were recruited from an ongoing study that applied the same methodology used for healthy adults, with the exclusion criteria of history of smoking, chronic respiratory disease, or hospitalization for COVID-19-induced pneumonia. We used propensity score matching to select the healthy, matched control group. This selection was based on demographic characteristics relevant to lung function outcomes, such as sex, age, height, and weight, from the control cohort. Table 1 presents data on the demographic and clinical characteristics of the patients.

**TABLE 1:** Anthropometric data and clinical characteristics of the patients involved in the study groups.

		Group COVID (*n* = 9)	Group H (*n* = 9)	*p*
Anthropometric data mean ± SD	Female/male (*n*)	4/5	4/5	1.0
	Age (years)	42.0 ± 11.1	45.0 ± 11.5	0.6
	Height (cm)	167 ± 9.4	172 ± 6.6	0.7
	Weight (kg)	91.6 ± 14.2	84.2 ± 7.4	0.18
	BMI (kg/m^2^)	32.7 ± 5.2	28.2 ± 2.7	0.14
Status mean (range)	Apache II score	14.4 (9–33)		
	LISS score	3.2 (3–3.5)		
	RESP score	4.9 (2–7)		
Length of stay mean (range)	Hospital (days)	58.3 (34–94)		
	Total ICU (days)	55.0 (24–94)		
	V-V ECMO (days)	33.1 (10–65)		
	Duration of mechanical ventilation	44 (11–79)		
Pre-ECMO management	Duration of mechanical ventilation before ECMO (days)	2.5 (1–6)		
	ICU before V-V ECMO (days)	7.1 (2–18)		
	Prone position (number)	7		
Pre-ECMO parameters mean (range)	PEEP (cmH_2_O)	9.3 (8–12)		
	Driving pressure (cmH_2_O)	21.4 (15–26)		
	VT (ml/kg)	7.8 (6–10.7)		
	Crs (ml/cmH_2_O)	26.3 (17–41)		
	FiO_2_ (%)	97.5 (90–100)		
	PaO_2_ (mmHg)	68.0 (42–90)		
	PaO_2_/FiO_2_ (mmHg)	69.8 (47–100)		
	PaCO_2_ (mmHg)	61.8 (44–84)		
	pH	7.31 (7.13–7.39)		
ECMO parameters	Mean gas flow (l/min)	4.4 (3.0–5.5)		
	Blood flow (l/min)	4.6 (3.0–5.9)		

Apache II score, acute physiology and chronic health evaluation score; LISS score, lung injury score; RESP score, respiratory ECMO prediction score; ICU, intensive care unit; V-V ECMO, veno-venous extracorporeal membrane oxygenation; FiO_2_, fraction of inspired oxygen; PEEP, positive end-expiratory pressure; VT, tidal volume; Crs, respiratory system compliance; PaO_2_, arterial oxygen tension; PaCO_2_, arterial carbon dioxide tension.

**FIGURE 1:**
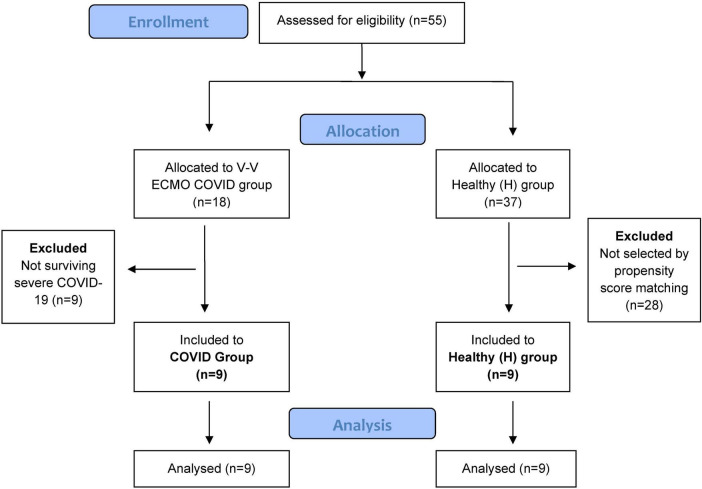
Patient flow chart. Altogether 55 patients were assessed for eligibility and were divided into the two study groups. We allocated 18 patients with severe acute COVID-19, all of whom received V-V ECMO support at our tertiary center. Nine patients in the COVID group were excluded as they did not survive, leaving nine post-COVID patients for the final analyses. Control group of patients were recruited from an ongoing study applying the same methodology as for healthy adults. Exclusion criteria included a history of smoking, chronic respiratory disease, or COVID-19-induced pneumonia requiring hospitalization. We selected 9 control subjects using propensity score matching, based on demographic characteristics relevant to lung function outcomes such as sex, age, height, and weight.

The current study protocol was approved by the Human Research Ethics Committee of University of Szeged, Hungary (*no. 143/2021 for the post-COVID patients and 186/2020 for the control group; Trial registration no. NCT05812196*). The patients provided a written informed consent. The study was performed in accordance the CONSORT guidelines. Figure 1 shows the patient flow chart.

### Mechanical ventilation and extracorporeal life support

Patients requiring V-V ECMO support received initially invasive ventilation. Based on the updated Extracorporeal Life Support Organization guidelines, V-V ECMO (20) was initiated using either Cardiohelp system with HLS Set Advanced (Getinge AB, Göteborg, Sweden) or Novalung combined with Xlung Patient Kit (Fresenius Medical Care, Bad Homburg, Germany). We used a 25 Fr, 38 cm long cannula for access, and a 19 to 25 Fr cannula with a length of either 15 or 55 cm for return, depending on the configuration. Regarding the technique, jugular-femoral cannulation was applied in 8 patients, and femoro-femoral cannulation was used in 1 patient. During V-V ECMO, lung protective ventilation was performed under pressure-control mode with the following parameters: FiO_2_, 40%; positive end-expiratory pressure, 10–15 cmH_2_O; driving pressure, 10 cmH_2_O; and respiratory rate, 10 cycles/min. The patients received unfractionated heparin with the following target levels: activated clotting time at 160–180 s or activated partial thrombin time at 46–55 s. Weaning from V-V ECMO was initiated if the tidal volume reached 4–6 mL/kg while maintaining a driving pressure of 10 cmH_2_O during pressure-controlled ventilation, and if the 100% oxygen test evidenced a significant contribution of the lungs to blood oxygenation.

### Primary outcome variables assessed by respiratory oscillometry

Respiratory oscillometry was used to measure the mechanical properties of the airways and the respiratory tissues. The technique is based on the introduction of small-amplitude pressure oscillations into the airway opening by using an external pressure generator (21). Recording of the oscillatory pressure (Pao) and airflow (V’) at various frequencies allows the calculation of the input impedance of the respiratory system, as Zrs = Pao/V’. Zrs data at each oscillatory frequencies can be expressed as a complex quantity represented by the respiratory resistance and reactance. Resistance expresses the oscillatory pressure in phase with the flow and reflects the resistive loss in the respiratory system. Reactance is defined as the oscillatory pressure component out of phase with oscillatory flow and demonstrates respiratory tissue elasticity at low oscillatory frequencies.

In the present study, Zrs was measured during spontaneous breathing with a pseudorandom forcing signal at a frequency range of 5–19 Hz (Resmon Pro Full system, Restech S.r.l, Italy). Measurements were performed while the patients were in an upright sitting position with cheeks supported in accordance with the European Respiratory Society (ERS) guidelines (22). Participants wore a nose clip, and they were instructed to breathe normally via a tightly sealed mouthpiece. At least three technically acceptable and reproducible 30-s long recordings were then performed. The impedance of the antibacterial filter was measured before each test, and this instrumental component was subtracted from the Zrs data.

The resistance values of the whole breath at 5 Hz (R_5_) and 19 Hz (R_19_) were extracted from the Zrs data for further analyses. Large and small airways contribute to the parameter R_5_, whereas R_19_ reflects mainly the airflow resistance of the central conducting airways with less influence from the smaller bronchi. Accordingly, subtracting R_19_ from R_5_ (R_5_–R_19_) reveals the contribution of the small airways to the overall airway resistance, with providing information on the ventilation inhomogeneities (12, 23). The area under the reactance curve from 5 Hz until the resonant frequency (AX_5_) represented the respiratory tissue stiffness (elastance). The resonant frequency (f_*res*_) at which Xrs crosses zero (where the elastic and inertial forces equilibrate with each other) was included in the data analyses.

### Spirometry

Spirometry was performed in accordance with the ATS/ERS guidelines (24). Forced expiratory flow-volume curves were measured with a commercially available spirometer (MasterScreen PFT, CareFusion, Höchberg, Germany). The flow signal was integrated to identify changes in lung volume during the forced expiratory maneuvers. Data on forced expiratory volume in the first second of expiration (FEV_1_), forced vital capacity (FVC), FEV_1_/FVC ratio, peak expiratory flow (PEF), and forced expiratory flow between 25 and 75% of the volume expired (FEF_25–75_) were extracted from the recordings. Three technically acceptable reproducible measurements were performed, and the highest values on spirometry were extracted from the maneuvers for the final analyses.

### Whole-body plethysmography

Functional residual capacity (FRC) and expiratory reserve volume (ERV) were measured via whole-body plethysmography (MasterScreen Body, Höchberg, Germany) using standard techniques established by the ERS/ATS Task Force (25).

### Measurement of alveolar gas diffusion

A single-breath method was used to evaluate the diffusing capacity of carbon monoxide (DLCO), carbon monoxide transfer coefficient (KCO), and alveolar volume (VA) (MasterScreen Diffusion, Höchberg, Germany).

### Data analyses

The reference values for the oscillometry outcomes were based on earlier established equations (26). The reference values of the parameters obtained via spirometry and gas diffusion were established according to the Global Lung Function Initiative Network guidelines (27). The measured values were reported as absolute values with scatter expressed as standard deviations, percentage predicted, and Z-score if applicable. Data normality was tested with the Shapiro–Wilk test. The independent *t*-tests were used to compare the measured variables.

Sample sizes were estimated to detect a clinically relevant 25% difference in one of the primary outcome parameters (AX_5_). This parameter was selected because restrictive dysfunction was mainly anticipated in patients with post-COVID-19 syndrome (13, 14), and was best reflected by the oscillometric parameters reflecting respiratory tissue stiffness. Accordingly, nine patients in the control and diseased groups were sufficient for detecting a statistically significant difference, with a variability of 10%, power of 80%, and a significance level of 5%. Propensity score matching was performed using the *MatchIt* package (version 4.4.0) (28) in the R software environment (version 4.2.1). Statistical tests were performed with the SigmaPlot statistical software package (version 13, Systat Software, Inc., Chicago, IL, USA), and a *p*-value of < 0.05 was considered statistically significant.

## Results

Regarding the clinical characteristics and anthropometric data of the COVID and healthy matched control groups, no significant differences were observed between the ground COVID and H in terms of female/male ratio, height, age, and body mass index (Table 1). Table 1 also presents the disease severity indices, duration of different interventions, and vital parameters obtained before initiating V-V ECMO support in the COVID group. Patients requiring V-V ECMO support received invasive ventilation for 0 to 10 days under pressure-controlled mode with specific ventilation parameters, as detailed in Table 1. None of the patients were smokers and none had chronic respiratory disease.

**FIGURE 2:**
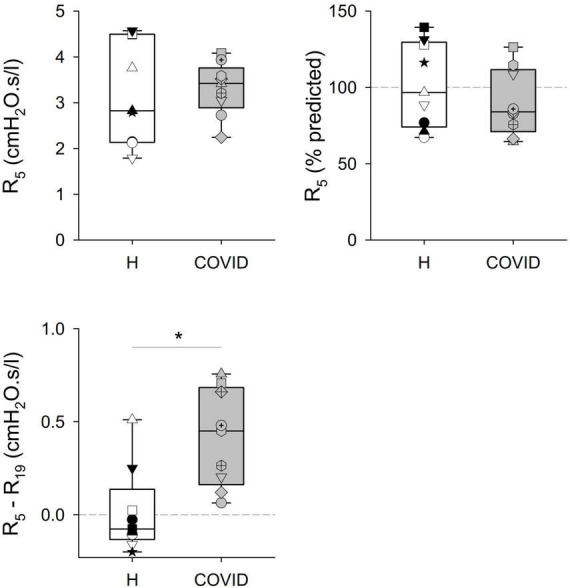
Mechanical parameters obtained via respiratory oscillometry characterizing airflow resistance at oscillation frequencies of 5 and 19 Hz (R_5_, R_19_), and their difference (R_5_–R_19_) reflecting the frequency dependence of the real part of the respiratory impedance spectra. Data were obtained at 6 months after hospital discharge in patients requiring veno-venous extracorporeal membrane oxygenation in the acute phase of coronavirus disease 2019 (COVID, gray shading) and in healthy matched controls (H, white shading). Data were reported as absolute values (**left** panels) and percent predicted (**right** panel), where the latter is available. Different symbols represent parameter values obtained in the individual patients. *p < 0.05 between the COVID-19 and healthy matched control groups.

**FIGURE 3:**
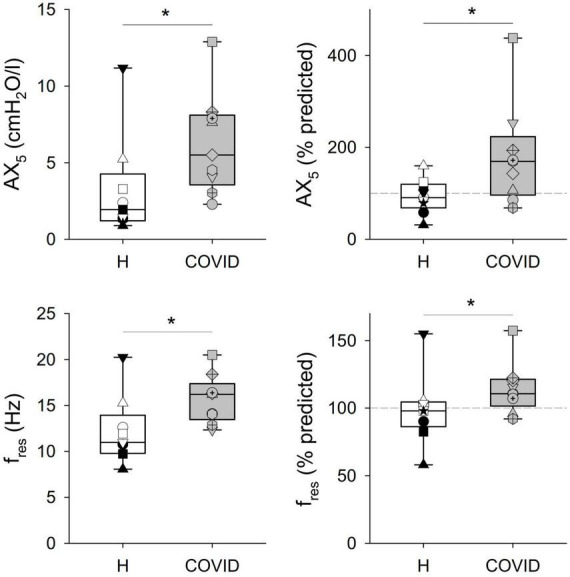
Respiratory tissue mechanical parameters obtained via respiratory oscillometry representing the area under the reactance curve at 5 Hz, and the resonant frequency (AX_5_) and the resonant frequency (f_*res*_). Data were obtained at 6 months after hospital discharge in patients requiring veno-venous extracorporeal membrane oxygenation in the acute phase of coronavirus disease 2019 (COVID, gray shading) and in healthy matched controls (H, white shading). Data were reported as absolute values (**left** panels) and percent predicted (**right** panels). *p < 0.05 between the COVID-19 and healthy matched control groups.

Significant differences were observed in some of the primary outcome variables reflecting the mechanical properties of the airways and the respiratory tissues between the two groups (Table 2 and Figures 2, 3). No statistically significant difference was observed in terms of R_5_ and R_19_ between the healthy matched control and COVID groups. Conversely, the COVID group had a significantly higher R_5_–R_19_ than in the control group. The difference in R_5_ and R_19_ was associated with a significantly higher AX_5_ and f_*res*_ in patients with COVID-19, with these differences remaining if these parameters are expressed as a percentage of predicted values or Z-scores.

**TABLE 2:** Mechanical parameters obtained via respiratory oscillometry characterizing airflow resistance at oscillation frequencies of 5 and 19 Hz (R_5_, R_19_), and their difference (R_5_–R_19_) reflecting the frequency dependence of the real part of the respiratory impedance spectra, area under the reactance curve at 5 Hz, and the resonant frequency (AX_5_) and the resonant frequency (f_*res*_).

		Group H	Group COVID	*p*
R_5_	Absolute value (cmH_2_O.s/l)	3.22 ± 1.13	3.31 ± 0.58	0.84
	% predicted	101.8 ± 27.7	89.9 ± 21.8	0.33
	Z score	-0.06 ± 0.94	-0.47 ± 0.91	0.36
R_19_	Absolute value (cmH_2_O.s/l)	3.21 ± 1.03	2.90 ± 0.40	0.42
	% predicted	106.5 ± 27.7	87.9 ± 18.6	0.12
	Z score	0.10 ± 0.93	-0.59 ± 0.85	0.12
R_5_–R_19_	Absolute value (cmH_2_O.s/l)	0.013 ± 0.23	0.411 ± 0.26	**0**.**003**
AX_5_	Absolute value (cmH_2_O/l)	3.20 ± 3.29	6.24 ± 3.31	**0**.**02**
	% predicted	93.99 ± 37.6	181.1 ± 111.9	**0**.**03**
	Z score	-0.21 ± 0.67	0.62 ± 0.79	**0**.**03**
f_*res*_	Absolute value (Hz)	12.2 ± 3.6	15.7 ± 2.6	**0**.**03**
	% predicted	99.1 ± 25.6	114.6 ± 18.9	**0**.**04**
	Z score	-0.24 ± 0.80	0.52 ± 0.65	**0**.**04**

Data were obtained at 6 months after hospital discharge in patients requiring veno-venous extracorporeal membrane oxygenation in the acute phase of coronavirus disease 2019 (Group COVID) and in healthy matched controls (Group H). Data were reported as absolute values, percent predicted, and Z-scores, where the latter two are available. Bold values: statistically significant difference.

The COVID group exhibited a significantly lower FEV_1_ and FVC than the healthy matched control group (Table 3). Due to the more severe decrease in FVC compared to FEV_1_, the COVID group showed a significantly higher FEV_1_/FVC ratio, expressed as absolute values, percentage predicted, or Z-scores. Meanwhile, there were no significant differences in terms of FEF_25–75_ or PEF between the healthy matched control and COVID groups.

**TABLE 3:** Lung function parameters obtained via spirometry at 6 months after hospital discharge in patients requiring veno-venous extracorporeal membrane oxygenation in the acute phase of coronavirus disease 2019 (Group COVID) and in healthy matched controls (Group H).

		Group H	Group COVID	*p*
FEV_1_	Absolute value (liters)	3.51 ± 0.89	2.68 ± 0.50	**0**.**01**
	% predicted	97.6 ± 7.5	79.5 ± 18.2	**0**.**02**
	Z score	-0.18 ± 0.56	-1.54 ± 1.28	**0**.**01**
FVC	Absolute value (liters)	4.39 ± 1.04	3.04 ± 0.45	**<0**.**001**
	% predicted	101.2 ± 7.8	73.4 ± 15.9	**<0**.**001**
	Z score	0.05 ± 0.67	-2.0 ± 1.18	**<0**.**001**
FEV_1_/FVC	Absolute value (%)	79.9 ± 4.7	87.9 ± 4.8	**0**.**001**
	% predicted	100.0 ± 4.9	114.1 ± 10.0	**0**.**05**
	Z score	0.02 ± 0.71	1.09 ± 0.69	**0**.**002**
FEF_25–75_	Absolute value (l/s)	3.34 ± 0.98	4.05 ± 1.51	0.24
	% predicted	93.2 ± 17.8	118.1 ± 41.5	0.12
	Z score	-0.27 ± 0.61	0.57 ± 1.49	0.14
PEF	Absolute value (l/s)	8.12 ± 1.43	7.50 ± 1.48	0.33
	% predicted	98.9 ± 10.3	96.9 ± 20.0	0.78
	Z score	-0.10 ± 0.7	-0.23 ± 1.44	0.81

FEV_1_, forced expiratory volume in 1 s; FVC, forced vital capacity; FEF_25–75_, forced expiratory flow between 25 and 75% of the volume expired; PEF, peak expiratory flow. Data were expressed as absolute values, percent predicted, and Z-scores. Bold values: statistically significant difference.

Regarding the diffusion capacity measurements, the COVID group had a significantly lower DLCO and VA, expressed as absolute or percentage predicted values than the healthy matched control group (Table 4). However, there was no difference in terms of KCO between the COVID and healthy matched control groups.

**TABLE 4:** Diffusing capacity of carbon monoxide (DLCO), alveolar volume (VA), and carbon monoxide transfer coefficient (KCO) measured at 6 months after hospital discharge in patients requiring veno-venous extracorporeal membrane oxygenation in the acute phase of severe coronavirus disease 2019 (Group COVID) and healthy matched healthy controls (Group H).

		Group H	Group COVID	*p*
DLCO	Absolute value (ml/min/mmHg)	26.7 ± 6.9	18.9 ± 4.3	**0**.**003**
	% predicted	90.0 ± 12.9	66.9 ± 14.5	**0**.**001**
VA	Absolute value (liters)	5.96 ± 1.2	4.11 ± 0.4	**<0**.**001**
	% predicted	94.9 ± 7.4	73.9 ± 15.8	**0**.**004**
KCO	Absolute value (1/min)	4.48 ± 0.69	4.56 ± 0.81	0.82
	% predicted	96.7 ± 11.5	94.1 ± 17.4	0.70

Data were presented as absolute values and percent predicted. Bold values: statistically significant difference.

As for the results using whole-body plethysmography, the significantly low FRC values obtained in the COVID group were associated with a remarkable decrease in ERV and its percentage predicted value (Table 5).

**TABLE 5:** Functional residual capacity (FRC) and expiratory reserve volume (ERV) measured using whole-body plethysmography at 6 months after hospital discharge in patients requiring veno-venous extracorporeal membrane oxygenation in the acute phase of severe coronavirus disease 2019 (Group COVID) and healthy matched healthy controls (Group H).

		Group H	Group COVID	*p*
FRC	Absolute value (liters)	3.22 ± 0.71	2.21 ± 0.30	**<0**.**001**
	% predicted	99.2 ± 15.6	73.0 ± 9.4	**<0**.**001**
ERV	Absolute value (liters)	1.33 ± 0.54	0.87 ± 0.28	**0**.**01**
	% predicted	104.0 ± 30.1	66.9 ± 211	**0**.**002**

Data were reported as absolute values and percent predicted. Bold values: statistically significant difference.

## Discussion

The main findings of this study demonstrate long-term detrimental pulmonary changes 6 months after hospital discharge, with deteriorations in the respiratory oscillometric parameters reflecting the frequency dependence of resistance (R_5_–R_19_) and the respiratory tissue stiffness (AX_5_). These adverse alterations in the oscillometric respiratory mechanical parameters were associated with reduced forced expiratory volumes (FEV_1_, FVC) and static lung volumes (VA, FRC, and ERV) in patients with post-COVID-19 syndrome. The adverse changes in lung function were reflected in reduced lung diffusion capacity (DLCO) without alterations in the carbon monoxide transfer coefficient (KCO).

An important feature of the current study is the ability to individually characterize the long-term effects of severe COVID-19 on the airway and respiratory tissue compartments. Resistance parameters obtained via respiratory oscillometry have the ability to characterize both overall and peripheral airway function, taking advantage of the fact that low-frequency oscillatory signals can reach even the small airways. Thus, this part of the oscillatory impedance reflects energy loss in the entire bronchial tree. Conversely, the proximal airways are mainly accessed by applying higher oscillatory frequencies. That is, these resistance components reflect central airway properties. Since R_19_ did not exhibit detrimental changes in patients with COVID-19, the mechanical properties of the large conducting airways were not affected by post-COVID-19 syndrome. On the contrary, the COVID group exhibited a significantly higher frequency dependence of respiratory resistance than the healthy matched control group, as evidenced by elevated R_5_–R_19_ data. This indicates the presence of a distal airway dysfunction at >6 months after severe COVID-19 infection, which is a result of heterogeneous peripheral airway constriction and/or permanent closure of terminal airspaces. These oscillometric findings are also supported by the results obtained via spirometry, thereby demonstrating a significant decrease in FVC. This dominant change affects the changes in other forced expiratory volumes and flow parameters. The decrease in FEV_1_ associated with a greater reduction in FVC results in an increased FEV_1_/FVC ratio in patients with COVID-19. This finding also suggests that the central conducting airways have normal function. FEF_25–75_ reflects small airway function; however, this parameter did not differ between the healthy matched control and COVID groups. This apparent controversy regarding oscillometric findings can also be attributed to a significant decrease in FVC without changes in PEF, which results in a preserved mid-expiratory flow in patients with post- COVID-19 syndrome.

To the best of our knowledge, there are no previous studies that have assessed the long-term changes in lung function in patients with severe COVID-19 requiring V-V ECMO support. The only publication in this clinical scenario is a case report focusing on radiological changes during V-V ECMO therapy (29). Accordingly, our findings can be compared with previous findings obtained in patients with post-COVID syndrome with various severity and time windows. In accordance with the findings of the current study, the dominance of peripheral airway dysfunction was observed in hospitalized non-ventilated patients with COVID-19 at 3 months after hospital discharge (30). The lack of remanent airway dysfunction was also found at 1-year follow-up among patients with post-COVID-19 syndrome. However, the involvement of a mixed population with only 24% of patients requiring invasive mechanical ventilation explains the discrepancy in our data (12). Furthermore, the decrease in FEV_1_ without a detrimental change in FEV_1_/FVC and PEF in our patients with post-COVID-19 syndrome is in agreement with previous results reported in similar clinical settings (7–9, 13, 14). A previous study also showed an involvement in persistent dysfunction in the conducting airways (11). However, older patients were included in these analyses, and smokers and participants with cardiopulmonary comorbidities were not excluded. These factors could explain the elevated low-frequency resistance and the abnormal FEV_1_/FVC ratio and FEF_25–75_.

Another important finding of the current study is the presence of persistent deterioration in respiratory tissue elastance, as reflected by the sustained elevations in AX_5_. No change in the resistive properties of the conducting large airways was detected, and the inertive forces remained unchanged. Therefore, the high f_*res*_ also reflects stiffer respiratory tissues in patients with post-COVID-19 syndrome compared to in healthy matched controls. This respiratory mechanical defect can be explained by two different mechanisms: a loss of lung volume leading to a stiffer working lung and intrinsic alteration in the respiratory tissues due to chronic remodeling. Our findings on the static lung volumes obtained via spirometry (FVC), plethysmography (FRC and ERV), and gas washout (VA) uniformly demonstrate the presence of persistent lung volume loss in patients with post-COVID-19 syndrome, thereby indicating the primary involvement of this mechanism in elevated respiratory tissue elastance. Regarding the potential additional effect of intrinsic changes in the respiratory tissues, our findings provide indirect evidence of the lack of tissue remodeling. Decreased DLCO, reflecting the overall gas-exchanging function of the whole lungs, was not associated with any change in KCO representing gas exchange per unit of lung volume. Since the changes in KCO were not statistically significant, these findings indicate the dominance of lung volume loss over lung tissue remodeling.

There are no earlier studies assessing long-term changes in lung tissue mechanics and related lung volume and gas exchange outcomes in patients with severe COVID-19 requiring V-V ECMO support during the acute phase of infection. Therefore, our findings can only be compared with previous data obtained from patients with COVID-19 requiring invasive ventilation and/or intensive care. Our results on the persistent stiffening of respiratory tissues are consistent with the high AX_5_ at 30 days and 3 months after hospital discharge, considering the changes in this elastance parameter (30). Due to the mechanisms responsible for this restrictive persistent lung mechanical defect, low static lung volumes have been consistently reported in patients with severe post-COVID-19 syndrome (7, 13, 31–35). This finding is similar to ours. The dominance of lung volume loss over the fibrotic lung tissue modeling according to the long-term effects of severe COVID-19 is also in agreement with previous results showing consistent decreases in DLCO (7, 10, 12, 13, 31, 32, 34, 36, 37) with preserved KCO (10, 12, 13, 32).

V-V ECMO is an acute, life-saving extracorporeal gas exchange support modality. However, it has several direct and indirect pulmonary consequences. In terms of the direct effects of V-V ECMO, it can facilitate protective lung ventilation possibly, by applying low driving pressure and tidal volume (VT) with low FiO_2_ and ventilation frequency. Conversely, the application of low VT may cause the development of persistent atelectasis, despite the maintenance of a relatively high positive end-expiratory pressure. In addition, the systemic inflammatory response induced by the pathogen may be further aggravated by indirect mechanisms related to the large artificial instrumental surface of the V-V ECMO. The resultant long-term effects of these pathophysiological processes are not completely understood. The respiratory outcomes of patients with severe COVID-19 requiring V-V ECMO support were comparable to those in earlier studies on patients with COVID-19 who presented with a more moderate disease severity, with (7, 8, 12, 13, 31, 35, 37) or without (7, 8, 12–14, 30, 31, 35–37) the need for invasive ventilation. Hence, the long-term pulmonary protective features may outweigh the temporary negative effects of V-V ECMO, which is associated with good health-related quality of life (38).

The current study had several limitations. At our institution, 18 patients with COVID-19 were treated with V-V ECMO; however, the survival rate of this cohort was 50%. This resulted in a relatively small sample size, allowing the recruitment of a maximum of nine patients discharged from the hospital. However, the power level of the statistical tests was sufficient to detect differences with confidence. Thus, conclusions are supported by the current datasets.

Patients who received V-V ECMO underwent highly invasive diagnostic and therapeutic procedures, supplemented by radiological imaging involving radiation exposure in the acute phase of COVID-19. Therefore, in this follow-up study, we aimed to apply techniques that are non-invasive and do not expose patients to ionizing radiation. If these non-invasive techniques promote sufficient recovery, invasive modalities may be considered to complete post-COVID-19 follow-up.

Another aspect of our work is related to the time window of 6 months after hospital discharge. Since adverse pulmonary effects (12, 15, 18) and worsening of health-related quality of life (16) persist following COVID-19 infections over a longer term, an extension of the study period beyond 6 months is planned to reveal temporal changes in the outcomes reported in the present study.

Since the present study focused on lung function outcomes 6 months after hospital discharge, a further limitation of our study is the lack of identification of biomarkers specific to ARDS (39). Accordingly, investigating the potential correlation between the biomarkers specific to endothelial and/or alveolar epithelial injuries in ARDS with post-COVID lung functional outcomes is a subject of further investigation.

## Conclusion

Patients with severe COVID-19 who required V-V ECMO support for severe respiratory failure during the acute phase of infection present residual pulmonary dysfunction. Compromised small airway function and loss of working lung volume are the dominant pathologies found 6 months after hospital discharge. Our findings emphasize the importance of thorough follow-up of lung function in patients required V-V ECMO support in the acute phase of COVID-19 infection, even 6 months after hospital discharge.

## Data availability statement

The raw data supporting the conclusions of this article will be made available by the authors, without undue reservation.

## Ethics statement

The studies involving humans were approved by the Human Research Ethics Committee of University of Szeged, Hungary (no. 143/2021 for the post-COVID patients and 186/2020 for the control group; Trial registration no. NCT05812196). The studies were conducted in accordance with the local legislation and institutional requirements. The participants provided their written informed consent to participate in this study.

## Author contributions

AP: Data curation, Formal analysis, Investigation, Methodology, Supervision, Writing – original draft, Writing – review and editing. ÁB: Conceptualization, Data curation, Formal analysis, Investigation, Methodology, Writing – original draft, Writing – review and editing. GP: Data curation, Formal analysis, Investigation, Methodology, Writing – review and editing. DS: Data curation, Formal analysis, Investigation, Methodology, Writing – review and editing. ÉZ: Conceptualization, Data curation, Investigation, Methodology, Supervision, Validation, Writing – review and editing. GB: Conceptualization, Data curation, Investigation, Methodology, Validation, Writing – review and editing. GF: Conceptualization, Data curation, Formal analysis, Investigation, Methodology, Supervision, Visualization, Writing – review and editing. KB: Data curation, Investigation, Methodology, Supervision, Validation, Writing – review and editing. AS: Conceptualization, Data curation, Investigation, Methodology, Project administration, Supervision, Validation, Writing – review and editing. FP: Conceptualization, Data curation, Formal analysis, Investigation, Methodology, Supervision, Validation, Writing – original draft, Writing – review and editing, Funding acquisition, Visualization. BB: Data curation, Formal analysis, Investigation, Methodology, Supervision, Writing – original draft, Writing – review and editing, Conceptualization, Validation.
